# Factors Associated with Depressive Symptoms in Korean Adults with Diabetes Mellitus: A Cross-Sectional Study

**DOI:** 10.3390/healthcare9081049

**Published:** 2021-08-16

**Authors:** Mihyun Jeong

**Affiliations:** Department of Nursing, Changshin University, Changwon 51352, Korea; mjeong@cs.ac.kr

**Keywords:** diabetes mellitus, depressive symptoms, Korean adults, Patient Health Questionnaire-9

## Abstract

Depressive symptoms in adults with diabetes are influenced by sociodemographic status, health-related behaviors, and comorbid diseases. This study aimed to examine the factors related to depressive symptoms in Korean adults with diabetes, using data from the Korea National Health and Nutrition Examination Surveys for 2014, 2016, and 2018. A total of 1529 Korean adults with diabetes were selected as subjects for the analysis. The age group of the participants was 19–80 years, with a mean age of 63.34 ± 0.68 years. The depressive symptoms and severity were assessed using the Korean version of the Patient Health Questionnaire-9. Descriptive statistics, chi-squared tests, and univariate and multivariable logistic regression analyses were used by applying a complex sample analysis method. The findings showed that 9.6% of Korean adults with diabetes exhibited moderate to severe depressive symptoms, which gradually decreased during 2014–2018. The most significant independent factors of depressive symptoms were living without a spouse, unemployment, low household income, fair or poor subjective health conditions, high perceived stress, a diabetes duration of over 20 years, and stroke. In females, living without a spouse, low household income, poor subjective health condition, high perceived stress, stroke, and coronary heart disease were significantly associated with depressive symptoms. In males, living without a spouse, unemployment, poor subjective health condition, high perceived stress, and hypertension were significantly associated with depressive symptoms. These findings highlight the importance of regular screening for depressive symptoms in patients with diabetes as the prevalence of depressive symptoms in people with diabetes may be higher than those in the general population. Future studies should also examine the development and effectiveness of psychosocial intervention programs to decrease depressive symptoms in patients with diabetes, considering cost-effective and time-saving approaches.

## 1. Introduction

Diabetes poses a major public health problem worldwide as its prevalence has increased steadily over the past few decades. Approximately 463 million adults, or one in 11 adults, lived with diabetes in 2019 [1,2]. Korea is no exception; in 2018, the prevalence of diabetes among Korean adults over 30 years of age was 13.8%, which has remained steady over the last seven years [3]. Depression, another debilitating condition, affects over 300 million people worldwide, according to a 2017 report [4]. In particular, depression is highly prevalent in people with diabetes and negatively affects their quality of life [5]. 24 studies have provided evidence for the comorbidity of diabetes and depression. Although the causal relationship between diabetes and depression remains unknown, studies have shown that the risk of developing depression is higher in people with diabetes [6]. A meta-analysis of 39 studies showed that the likelihood of depression in people with diabetes was twice that of people without diabetes [7]. Approximately 10–20% of people with diabetes experience depressive symptoms and are more likely to have a moderate to severe form of depression [8,9,10,11]. The high burden of diabetes complications and diabetes itself can increase the risk of depressive symptoms. As a result, comorbid depressive symptoms can aggravate the symptoms and complications of diabetes. An epidemiological study reported that people with diabetes and depressive symptoms had twice as many complications and symptoms of diabetes, compared with those without diabetes [12].

Clinically, the presence of depression may adversely impact the short- and long-term control of diabetes. For example, according to a report [13], people with diabetes who displayed depressive symptoms had higher levels of glycated hemoglobin (A1C) than individuals without depressive symptoms, as depressive symptoms have a negative effect on treatment adherence [14] and diabetes self-management, including appropriate diet and physical activity [15]. Further, diabetes leads to increased healthcare utilization and high medical costs [16]. A large population-based study demonstrated that comorbidity and physical impairment were correlated with depression in people with diabetes [17]. Additionally, a meta-analysis of 14 cross-sectional studies reported that female sex, insulin use, diabetes complications, low educational level, physical inactivity, living alone, and unemployment were significant risk factors for depressive symptoms in people with diabetes [18]. A community-based epidemiological study investigating 3540 Korean adults with type 2 diabetes in a city identified low income, unemployment, current smoking, use of oral hypoglycemic medications or insulin, and physical inactivity as significant factors associated with depressive symptoms [19]. Although it is widely known that females are more likely to suffer from depressive symptoms than males [20], sex differences in factors associated with depressive symptoms in people with diabetes are not well known.

Given the pervasive impact of depressive symptoms on the progress of diabetes, the early identification of high-risk individuals and the effective management of comorbidities are warranted and require a better understanding of the factors associated with depressive symptoms in people with diabetes.

In Korea, most studies on depression have investigated the general population [21,22] or older adults [23]. Population-based studies on the association between depressive symptoms and diabetes are scarce. The Korean National Health and Nutrition Examination Survey (KNHANES) is a nationwide cross-sectional survey conducted annually by the Korea Centers for Disease Control and Prevention (KCDC) to estimate the health and nutrition of the Korean population. Assessment of depressive symptoms in the KNHANES has been conducted using the Patient Health Questionnaire-9 (PHQ-9)**,** a validated instrument, every 2 years since 2014.

Therefore, this study, using a dataset from a nationally representative survey of the KNHANES 2014, 2016, and 2018, aimed to examine the prevalence of depressive symptoms in Korean adults with diabetes and compare the sociodemographic, health behavioral, and diabetes-related characteristics of individuals with depressive symptoms with those individuals without depressive symptoms (Aim 1). We also explored the significant independent factors associated with depressive symptoms in Korean adults with diabetes and sex differences in factors related to depressive symptoms (Aim 2).

## 2. Materials and Methods

### 2.1. Study Sample

We conducted a descriptive, cross-sectional study using secondary data from the KNHANES for 2014, 2016, and 2018. The KNHANES was approved by the Institutional Review Board of The KCDC (No. 2013-12EXP-03-5C & 2018-01-03-P-A) and received written informed consent from all participants. The data and detailed information for the surveys are publicly available on the KCDC website (https://knhanes.kdca.go.kr, accessed on 13 August 2021). The KNHANES employs a complex sample design with a two-stage stratified cluster probability sampling method. Professional research teams conducted health behavior and nutrition surveys and a physical examination through face-to-face interviews in a mobile health vehicle.

The data of 1655 adults aged 19 years or older who were diagnosed with diabetes were extracted from the KNHANES for 2014, 2016, and 2018. Participants with incomplete data on their depressive symptoms were excluded from this study (*n* = 95, 5.74%). Moreover, we excluded 31 individuals with missing values on household income (*n* = 10), physical activity (*n* = 10), monthly drinking (*n* = 3), perceived stress (*n* = 3), and body mass index (BMI) (*n* = 5). Consequently, a sample of 1529 adults with diabetes mellitus was included in the analysis.

In this study, the presence of diabetes in the respondents was assessed based on a self-reported questionnaire asking participants to disclose if they had been diagnosed with diabetes by a physician, as self-perceived diabetes itself affects depressive symptoms [24].

### 2.2. Measurements

#### 2.2.1. Depressive Symptoms

Depressive symptoms were measured using the Korean version of the Patient Health Questionnaire-9 (PHQ-9) as a screening tool for two weeks. It is a brief validated evaluation of the major depressive disorders [25,26]. The PHQ-9 is based on the Diagnostic and Statistical Manual of Mental Disorders, Fourth Edition, criteria for major depressive disorder, containing nine items depicting depressive symptoms. The PHQ-9 is a screening tool to evaluate the presence of depressive symptoms over the past two weeks. The total score was between 0 and 27, and each item was scored from 0 (“not at all”) to 3 (“nearly every day”). The PHQ-9 score was divided into 5–9, 10–14, 15–19, and 20 or above, indicating mild, moderate, moderately severe, and severe symptoms. A score of 10 or more indicated clinically significant depressive symptoms [27], with a specificity of 80% and a sensitivity of 92% [28].

#### 2.2.2. Sociodemographic Characteristics

Sociodemographic characteristics included age, sex, education level, living area, living arrangement, employment, and household income. Age was categorized as 19–49, 50–59, 60–69, and ≥70 years. Education level was categorized as six years or less (elementary school), 7th–9th class (middle school), 10th–12th class (high school), and >12 years (college and beyond). The living area was classified as rural or urban, and the living arrangement was with or without a spouse. Employment status was divided into employed and unemployed groups. Finally, household income was categorized as quartiles after dividing the total household monthly income by the square root of the household number.

#### 2.2.3. Health Behavioral Characteristics

Health behavioral characteristics included current smoking, monthly drinking, subjective health conditions, perceived stress, physical activity, and Body Mass Index (BMI). The former two were classified as a yes or no. Subjective health conditions were categorized as good, fair, or poor. Perceived stress was categorized as low and high levels. Finally, physical activity was measured using the Korean version of the Global Physical Activity Questionnaire, a validated instrument developed by the World Health Organization (WHO) [29]. Physical activity was determined as 150 min of moderate or 75 min of vigorous intensity weekly or an equivalent combination. It was classified as low (inactive) and moderate to vigorous (activity) levels. Based on a previous study, body weight and height were measured to calculate the BMI (body weight divided by height squared). According to the WHO’s criteria for the Asia-Pacific region [30], BMI was classified as underweight (<18.5 kg/m^2^), normal (18.5–22.9 kg/m^2^), overweight (23.0–24.9 kg/m^2^), and obese (≥25.0 kg/m^2^).

#### 2.2.4. Diabetes-Related Characteristics

Diabetes duration, management of diabetes, A1C, and comorbid diseases were collected as data for diabetes-related characteristics. Diabetes duration was classified into one year or less, 2–5 years, 6–10 years, 11–20 years, and over 20 years. Management of diabetes was grouped into no treatment, no medication, only insulin, only oral hypoglycemic agent (OHA), and both OHA and insulin. A1C was measured using the Tosoh G8 high-performance liquid chromatography (Tosoh, Tokyo, Japan) with venous blood samples. It was classified to less than 7% and 7% or more, indicating good glycemic control and poor glycemic control, respectively [31]. Comorbidities related to diabetes complications included hypertension, dyslipidemia, stroke, and coronary heart disease [32], and the presence of self-reported comorbid diseases was categorized as a yes or no.

#### 2.2.5. Outcomes

This study’s dependent variable was depressive symptoms. Independent variables included diabetes-related variables (diabetes duration, management of diabetes, A1C, and comorbid diseases), health behavioral variables (current smoking, monthly drinking, subjective health conditions, perceived stress, physical activity, and BMI), and sociodemographic variables (age, sex, education level, living area, living arrangement, employment, and household income).

### 2.3. Statistical Analysis

Data collected from the KNHANES 2014, 2016, and 2018 were analyzed using a complex sample analysis method. Sampling weights, clustering, and stratification were applied to analyze the data and estimate the results for the target population. For Aim 1, descriptive statistics were computed for sociodemographic, health behavioral, and diabetes-related variables, including means and standard deviations (SD) for continuous variables, and frequencies and percentages for categorical variables. Chi-squared tests were used to compare the prevalence of depressive symptoms by severity every two years from 2014 to 2018 and categorical variables of participants with and without depressive symptoms. According to Hosmer, Lemeshow, and Sturdivant [33], logistic regression analyses included significant variables of *p* < 0.20 from the chi-squared tests and were described using odds ratios (ORs) and 95% confidence intervals (CIs). For Aim 2, we conducted univariate logistic regression analyses to examine the association of each of the potential factors related to depressive symptoms. Multivariable logistic regression analyses identified factors related to depressive symptoms in adults with diabetes. Based on the results of the chi-squared tests to compare each variable and participants with and without depressive symptoms by sex, multivariable logistic regression analyses were further performed to identify independent factors associated with depressive symptoms. The Hosmer–Lemeshow goodness-of-fit tests revealed good calibrations for the multiple logistic regression (χ^2^_(8)_ = 3.963, *p* = 0.861), female (χ^2^_(8)_ = 6.790, *p* = 0.559), and male (χ^2^_(8)_ = 6.865, *p* = 0.551) models. The Statistical Package for the Social Sciences version 26.0 (IBM/SPSS) software was used for all statistical analyses. Statistical significance was set at *p* < 0.05.

## 3. Results

A total of 1529 Korean adults with diabetes were included in the analysis. The mean age of the participants was 63.34 ± 0.68 years (range: 19–80 years). The participants were 47.2% female and 52.8% male.

### 3.1. Prevalence of Depressive Symptoms

**Figure 1:**
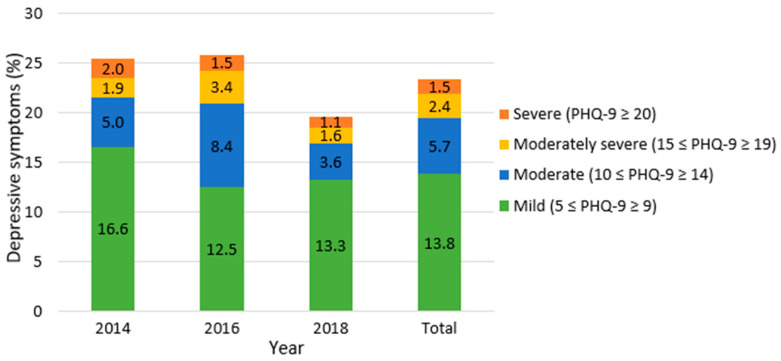
Prevalence of depressive symptoms by year.

Figure 1 describes the prevalence of Korean adults with diabetes who had depressive symptoms (classified by severity) in 2014, 2016, 2018, and overall. Overall, 9.6% (149) of participants reported depressive symptoms (PHQ-9 score ≥ 10). Mild symptoms were reported by 13.8 % (PHQ-9 score 5–9), 5.7% had moderate symptoms (PHQ-9 score 10–14), 2.4% had moderately severe symptoms (PHQ-9 score 15–19), and 1.5% had severe depressive symptoms (PHQ-9 score ≥ 20). Moreover, the prevalence of depressive symptoms (PHQ-9 score ≥ 5) in 2014 and 2016 was 25.5% and 25.8%, respectively. However, this score decreased significantly to 19.6% in 2018. Interestingly, the prevalence of severe symptoms gradually decreased from 2014 to 2018 (*p* < 0.05), and the proportion of participants reporting mild symptoms decreased between 2014 and 2016. However, this again increased slightly in 2018.

### 3.2. Differences in Sociodemographic Characteristics with and without Depressive Symptoms

Table 1 displays the sociodemographic characteristics of the study population, distributed by depressive symptoms. Participants with depressive symptoms were more likely to be a female (13.6%, *p* < 0.001), with elementary school education or lower (13.5%, *p* = 0.003), living without a spouse (17.3%, *p* < 0.001), unemployed (14.3%, *p* < 0.001), and with low household income (15.5%, *p* < 0.001), compared with those without depressive symptoms. Although the rate of depressive symptoms increased with age, there were no statistically significant differences in age and the living area between patients with and without depressive symptoms (*p* = 0.776 and 0.894).

### 3.3. Differences in Health Behavioral Characteristics with and without Depressive Symptoms

Table 2 presents the differences in health behavioral characteristics between patients with and without depressive symptoms. Significant group differences existed in monthly drinking, subjective health conditions, and perceived stress. For example, participants with depressive symptoms were more likely to report no monthly drinking (12.0%, *p* = 0.003), poor subjective health condition (20.2%, *p* < 0.001), and high perceived stress (27.0%, *p* < 0.001). However, the difference between the groups was not significant for current smoking, physical activity, and BMI.

### 3.4. Differences in Diabetes-Related Characteristics with and without Depressive Symptoms

Table 3 shows diabetes-related characteristics of the study population, distributed by depressive symptoms. We found significant between-group differences in diabetes duration and comorbid diseases, including hypertension, stroke, and coronary heart disease. Participants with depressive symptoms displayed a longer diabetes duration (*p* < 0.001), presence of hypertension (*p* = 0.024), presence of stroke (*p* < 0.001), and presence of coronary heart disease (*p* = 0.001). Conversely, no between-group differences existed in the management of diabetes, A1C, and the presence of dyslipidemia.

### 3.5. Factors Associated with Depressive Symptoms

Table 4 summarizes the analyses of the factors associated with depressive symptoms in Korean adults with diabetes. In the univariate analysis, sociodemographic factors (including being female, lower educational level (7–9 or ≤6 years), living without a spouse, unemployment, and low–medium or low household incomes), health behavioral factors (such as no monthly drinking, fair or poor subjective health conditions, and high perceived stress), diabetes-related factors (including diabetes duration of 20 years or more and use of both OHA and insulin), and the presence of comorbid diseases (such as hypertension, stroke, and coronary heart disease) were significantly associated with an increased risk of depressive symptoms.

In the multivariable logistic regression model, only the following variables remained significant factors of depressive symptoms: living without a spouse, unemployment, low household income, fair or poor subjective health conditions, high perceived stress, more than 20 years of diabetes duration, and presence of stroke. For example, participants living without a spouse had 2.05 (95% CI = 1.30–3.24) times higher odds of depressive symptoms than those living with a spouse. Compared with employed participants, unemployed participants had a 2.05 (95% CI = 1.11–3.77) times higher risk of depressive symptoms. Low household income was significantly associated with an increased risk of depressive symptoms (OR = 2.95, 95% CI = 1.30–6.69). In health behavioral variables, depressive symptoms were significantly higher in fair (OR = 5.21, 95% CI = 1.05–25.90) or poor (OR = 19.07, 95% CI = 4.11–88.39) subjective health conditions compared with good subjective health condition. Participants with high perceived stress showed a 6.98 (95% CI = 4.27–11.40) times greater risk of depressive symptoms. In diabetes-related variables, adults with a diabetes duration of 20 years or more had 3.89 (95% CI = 1.09–13.95) times higher odds of exhibiting depressive symptoms than those with less than a year. As a comorbid disease, participants with stroke had 1.94 (95% CI = 1.03–3.67) times greater odds of depressive symptoms than those without stroke.

### 3.6. Factors Associated with Depressive Symptoms by Sex

Table 5 describes the sex-specific analyses of the factors associated with depressive symptoms. For female participants, living without a spouse (OR = 1.74, 95% CI = 1.03–2.92), low household income (OR = 4.33, 95% CI = 1.22–15.37), poor subjective health condition (OR = 16.76, 95% CI = 2.04–137.32), high perceived stress (OR = 4.69, 95% CI = 2.48–8.89), the presence of stroke (OR = 2.25, 95% CI = 1.03–4.91), and the presence of coronary heart disease (OR = 2.47, 95% CI = 1.14–5.38) showed an association with depressive symptoms. For male participants, living without a spouse (OR = 3.22, 95% CI = 1.23–8.44), unemployment (OR = 3.19, 95% CI = 1.31–7.75), poor subjective health condition (OR = 25.75, 95% CI = 3.05–217.49), high perceived stress (OR = 11.09, 95% CI = 4.95–24.88), and the presence of hypertension (OR = 8.28, 95% CI = 2.72–25.21) were statistically significantly associated with depressive symptoms. On the other hand, being underweight (OR = 0.07, 95% CI = 0.01–0.69) and overweight (OR = 0.26, 95% CI = 0.09–0.71) were significantly associated with a decreased risk of depressive symptoms.

## 4. Discussion

This study performed secondary analyses of the KNHANES 2014, 2016, and 2018 datasets to examine the factors associated with depressive symptoms and their prevalence in Korean adults with diabetes. Our findings indicate that 9.6% of participants exhibited depressive symptoms (PHQ-9 score ≥ 10). There was a gradual decrease in the prevalence of severe depressive symptoms (PHQ-9 score ≥ 20) between 2014 and 2018. By contrast, the mild depressive symptoms decreased between 2014 and 2016, and increased slightly in 2018. In other words, Korean adults with diabetes showed a decrease in the prevalence of severe symptoms and an increase in the prevalence of mild symptoms in 2018, compared with 2016. This difference may be due to early screening and ongoing care for depressive symptoms as potential comorbidity in patients with diabetes in clinical practice.

This study’s results show that the prevalence of depressive symptoms is relatively higher among patients with diabetes than the general population. A national study of the general US adult population revealed that individuals with a PHQ-9 score ≥ 10 were 8.0% between 2015 and 2018 [34]. A cross-sectional study of the northeast Chinese population reported that the prevalence of depressive symptoms was 6%, using the PHQ-9 [35]. Similarly, a Korean population-based study using KNHANES 2014 and 2016 reported 6.1% [22]. Conversely, this study’s prevalence of depressive symptoms was relatively lower than previous studies conducted with diabetic adults in other countries. An international cross-sectional study conducted with the PHQ-9 in 21 developing countries reported moderate to severe depressive symptoms in 10.2% and 11.1% in adults with type 1 and type 2 diabetes, respectively [8]. In Indonesia, a cross-sectional survey using the PHQ-9 identified that 11.6% of adults with diabetes exhibited depressive symptoms (PHQ-9 score ≥ 10) [36]. A population-based study using the PHQ-9 among US adults with diabetes reported that 12% of participants suffered from major depression [37]. A cross-sectional study of Chinese adults with type 2 diabetes reported a prevalence of 35.1% using the Zung Self-rating Depression Scale [38]. A cross-sectional study using the Hospital Anxiety and Depression Scale in Irish adults with diabetes showed 10.1% of participants exhibiting moderate to severe depression [39]. To sum up, the present study highlights the high prevalence of depressive symptoms in adults with diabetes than the general population. The prevalence of depression in Korean adults with diabetes may be relatively lower than in other countries. Nevertheless, because different studies have used various tools to measure depressive symptoms, a direct comparison requires careful attention.

This study also identified independent factors associated with depressive symptoms in adults with diabetes. Sociodemographic factors such as living without a spouse, unemployment, and low household income were significant factors. This finding corroborates the findings of a systematic review that investigated factors related to depression in patients with diabetes [9]. On the other hand, a cross-sectional study including adults with type 1 and 2 diabetes reported that living status and employment were not associated with depressive symptoms [39]. A national-population study of patients with diabetes showed that sex, poor education, and low income were independent factors to major depressive disorder [40]. However, in this study, female sex and inferior education’s association with depressive symptoms were significant in the univariate analyses. Although, the literature reviews investigating the association between depression and diabetes have shown these as risk factors for depression [18,41]

Regarding health-related behavioral factors, fair and poor subjective health conditions and high perceived stress were significantly associated with depressive symptoms. In particular, poor subjective health conditions and high perceived stress significantly increased the risk of depressive symptoms. This risk may be because of poor glycemic control, poor diabetes self-management, and the psychological burden of diabetes complications leading to low self-esteem. These results confirm previous studies on factors of depressive symptoms both in the general population [21,42,43] and in adults with diabetes [44,45]. No monthly drinking was found to be associated with depressive symptoms in the univariate analysis.

In diabetes-related variables, diabetes duration of 20 years or more and stroke were at increased risk of depressive symptoms. Researchers have associated more prolonged diabetes and comorbid conditions with depressive symptoms [46,47,48]. A plausible explanation is that people with longer diabetes history have more comorbid diseases, such as diabetes complications, because of the chronic and progressive nature of the disease. During this process, people with diabetes may suffer from depressive symptoms [49]. This finding aligns with those found in a prospective cohort study associating depression with risks for advanced diabetes complications [12] and a systematic review of the relationship between depression and type 2 diabetes [46]. A systematic and meta-analysis of cohort studies investigating comorbid depression and the risk of cardiac diseases and mortality in people with diabetes revealed a significantly increased risk of cardiovascular mortality, coronary heart disease, and stroke [50]. A cross-sectional study including data of adults with type 1 and 2 diabetes reported that diabetes complications were an independent predictor of severe depressive symptoms [39]. In another cross-sectional study separately analyzing data for adults with type 1 and type 2 diabetes, cardiovascular disease was not associated with depressive symptoms in adults with either type 1 or type 2 diabetes, while proliferative retinopathy in adults with type 1 diabetes and neuropathy in adults with type 2 diabetes were significant predictors of depressive symptoms [48].

Lastly, this study determined independent factors associated with depressive symptoms by sex. Living without a spouse, poor subjective health condition, and high perceived stress were common major factors associated with depressive symptoms in both sexes. Low household income, stroke, and coronary heart disease for female adults and unemployment and hypertension for male adults were significantly associated with depressive symptoms. Among middle-aged and older Korean adults, women generally tend to take care of housework and children or parents, while men tend to be responsible for the household income. In this aspect, female adults with lower household incomes and unemployed male adults are more likely to develop depressive symptoms. The findings of the current study are partially consistent with the results of previous studies. A community study of older adults reported wealth for men and income for women as independent factors in the association between socioeconomic status and depressive symptoms [51]. Further, a cross-sectional study examining gender differences in depression in 23 European countries showed that living without a partner and low socioeconomic status for both men and women had strong associations with depression [20].

This study is the first to measure the prevalence of depressive symptoms using the PHQ-9 as a validated tool to analyze recent data from a nationally representative sample of Korean adults with diabetes. However, this study has some limitations. First, we assessed diabetes based on a self-reported measure rather than fasting plasma glucose and A1C tests because this study was focused on adults who were aware of having diabetes. Second, the cross-sectional nature of this study limits inferences about causalities between the associated factors and depressive symptoms. Third, we could not differentiate between adults with type 1 diabetes and type 2 diabetes, as it used secondary data from the KNHANES for the years 2014, 2016, and 2018. Although diabetes type is not a significant predictor of depressive symptoms [39], factors related to depressive symptoms may differ depending on diabetes type [48]. Fourth, as the present study did not contain all potential factors that influence depressive symptoms in people with diabetes, it is necessary to conduct further research within the context of the predictors considered. Fifth, only community-based adults with diabetes participated in this study, whereas hospitalized patients with more severe depressive symptoms were omitted. Therefore, depressive symptoms may have been underestimated.

## 5. Conclusions

In conclusion, although perhaps relatively lower than other countries, the prevalence of depressive symptoms in Korean adults with diabetes may be higher than that in the general population. The results show that living without a spouse, unemployment, low household income, high perceived stress, fair or poor subjective health condition, more than 20 years of diabetes duration, and stroke have independent and significant effects on depressive symptoms in adults with diabetes. Furthermore, the findings reveal significant sex differences in the factors related to depressive symptoms in adults with diabetes. Based on this study’s findings, it is essential to regularly screen for depressive symptoms in people with diabetes, especially in patients living without a spouse and with high perceived stress and poor subjective health, in males who are unemployed and have hypertension, and in females with low income, stroke, and coronary heart disease. It is equally imperative to identify individuals with diabetes and depression through effective and tailored intervention programs.

The KCDC has provided evidence-based recommendations for primary care physicians regarding accurate screening and diagnosis of depression in adults as well as appropriate treatment and management of depression. It underlines regular screening tests for depression with the PHQ-9 in adults with diabetes for early detection and accurate screening. The KCDC also recommends following the DSM-5 criteria for diagnosis of depression and considering other conditions such as functional status, medical history, past treatment history, and family history. Moreover, the recommendations for depression include efficient management of depression through non-pharmacological therapy such as lifestyle modification and patient education, depression management and prevention of exacerbation through effective drug treatment, and referral criteria to a mental health professional. Nevertheless, the high prevalence of comorbid depression in adults with diabetes may be due to the absence of appropriate and integrated health care systems, which leads to worse health outcomes for both diabetes and depression.

Therefore, health care professionals should play a pivotal role in the early detection and education of developing depressive symptoms through integrative assessment and a systematic approach in adults with diabetes; they should also consider sex differences in factors associated with depressive symptoms. Screening for depression should be conducted in conjunction with appropriate and comprehensive systems to ensure accurate diagnosis, effective treatment, and appropriate follow-up. Furthermore, sufficient treatment time, a professional workforce to provide self-care support and care coordination, an appropriate health care environment, and support for national health policies should be ensured for application and activation of integrated health care systems. In other words, collaborative care should use a systematic, multicomponent, and team-based approach to improve the quality and outcomes of care for people with comorbid diabetes and depression.

Research is needed to assess barriers to establishing integrative health care systems and how these barriers can be addressed. In addition, future studies should explore potential factors related to depressive symptoms according to sex and examine the development and effectiveness of psychosocial intervention programs to decrease depressive symptoms in patients with diabetes in both primary and specialty care settings.

## Figures and Tables

**Table 1:** Differences in sociodemographic characteristics by depressive symptoms (*n* = 1529).

Variables	Total	Without Depressive Symptoms (PHQ < 10)		With Depressive Symptoms (PHQ ≥ 10)		*p*-Value
		(*n* = 1380)		(*n* = 149)		
	*n* (%)	*n*	W (%)	*n*	W (%)	
Total	1529 (100%)	1380	90.4	149	9.6	
Sex						<0.001
Male	749 (52.8)	699	93.9	50	6.1	
Female	780 (47.2)	681	86.4	99	13.6	
Age (years), Mean ± SD	63.34 ± 0.68	63.04 ± 0.42		63.64 ± 1.29		0.776
19–49	127 (12.6)	116	90.2	11	9.8	
50–59	281 (25.1)	258	92.0	23	8.0	
60–69	487 (29.4)	436	89.8	51	10.2	
≥70	634 (32.9)	570	89.7	64	10.3	
Educational level (years)						0.003
≤6 (Elementary school)	665 (38.2)	578	86.5	87	13.5	
7–9 (Middle school)	260 (16.5)	237	90.8	23	9.2	
10–12 (High school)	390 (29.1)	361	91.8	29	8.2	
>12 (College and beyond)	214 (16.2)	204	96.3	10	3.7	
Living area						0.894
Rural	362 (20.6)	325	90.1	37	9.9	
Urban	1167 (79.4)	1055	90.4	112	9.6	
Living						<0.001
With spouse	1087 (72.5)	1006	93.3	81	6.7	
Without spouse	442 (27.5)	374	82.7	68	17.3	
Employment						<0.001
Employed	678 (50.3)	645	95.0	33	5.0	
Unemployed	851 (49.7)	735	85.7	116	14.3	
Household income						<0.001
Low	436 (29.5)	368	84.5	68	15.5	
Low–medium	382 (23.6)	345	89.4	37	10.6	
Medium–high	373 (23.7)	344	93.3	29	6.7	
High	338 (23.2)	323	95.8	15	4.2	

Note: *n* = unweighted sample size; W = weighted percentage; SD = standard deviation; PHQ = Patient Health Questionnaire.

**Table 2:** Differences in health behavioral characteristics by depressive symptoms (*n* = 1529).

Variables	Total	Without Depressive Symptoms (PHQ < 10)		With Depressive Symptoms (PHQ ≥ 10)		*p*-Value
		(*n* = 1380)		(*n* = 149)		
	n (%)	*n*	W (%)	*n*	W (%)	
Current smoking						0.267
Yes	272 (20.7)	240	88.3	32	11.7	
No	1257 (79.3)	1140	90.9	117	9.1	
Monthly drinking						0.003
Yes	634 (45.9)	587	93.2	47	6.8	
No	895 (54.1)	793	88.0	102	12.0	
Subjective health condition						<0.001
Good	221 (13.7)	219	99.3	2	0.7	
Fair	696 (47.1)	669	96.6	27	3.4	
Poor	612 (39.2)	492	79.8	120	20.2	
Perceived stress						<0.001
Low	1204 (78.2)	1139	95.2	65	4.8	
High	325 (21.8)	241	73.0	84	27.0	
Physical activity						0.794
Low	982 (61.1)	881	90.1	101	9.9	
Moderate to vigorous	547 (38.9)	499	91.2	48	8.8	
BMI (kg/m^2^), Mean ± SD	25.23 ± 0.18	25.04 ± 0.11		25.42 ± 0.36		0.133
Underweight (<18.5)	13 (0.7)	11	94.7	2	5.3	
Normal (18.5 to 22.9)	660 (42.4)	590	89.7	70	10.3	
Overweight (23.0 to 24.9)	607 (40.7)	546	90.5	61	9.5	
Obesity (≥25.0)	249 (16.3)	233	94.0	16	6.0	

Note: *n* = unweighted sample size; W = weighted percentage; SD = standard deviation; PHQ = Patient Health Questionnaire; BMI = body mass index.

**Table 3:** Differences in diabetes-related characteristics by depressive symptoms (*n* = 1529).

Variables	Total	Without Depressive Symptoms (PHQ < 10)		With Depressive Symptoms (PHQ ≥ 10)		*p*-Value
		(*n* = 1380)		(*n* = 149)		
	*n* (%)	*n*	W (%)	*n*	W (%)	
Diabetes duration (years), Mean ± SD	11.33 ± 0.60	9.26 ± 0.58		13.39 ± 1.04		<0.001
≤1	104 (7.3)	99	95.8	5	4.2	
2–5	427 (29.9)	389	91.6	38	8.4	
6–10	336 (22.8)	300	90.9	36	9.1	
11–20	441 (26.6)	405	91.5	36	8.5	
>20	221 (13.4)	187	81.5	34	18.5	
Management of diabetes						0.065
Only OHA	1294 (84.0)	1176	91.3	118	8.7	
Only insulin	22 (1.3)	18	78.1	4	21.9	
OHA and insulin	97 (6.2)	84	82.7	13	17.3	
No medication	6 (0.3)	5	84.6	1	15.4	
No treatment	110 (8.2)	97	88.3	13	11.7	
A1C (%), *n* = 1415, Mean ± SD	7.21 ± 0.07	7.25 ± 0.04		7.16 ± 0.14		0.843
< 7.0	722 (50.1)	650	90.8	72	9.2	
≥7.0	693 (49.9)	631	90.5	62	9.5	
Comorbid diseases						
Hypertension						0.024
Yes	980 (60.6)	874	88.7	106	11.3	
No	549 (39.4)	506	92.9	43	7.1	
Dyslipidemia						0.482
Yes	735 (47.7)	656	89.7	79	10.3	
No	794 (52.3)	724	91.0	70	9.0	
Stroke						<0.001
Yes	110 (7.0)	85	76.3	25	23.7	
No	1419 (93.0)	1295	91.4	124	8.6	
Coronary heart disease						0.001
Yes	141 (8.0)	114	81.2	27	18.8	
No	1388 (92.0)	1266	91.1	122	8.9	

Note: *n* = unweighted sample size; W = weighted percentage; SD = standard deviation; PHQ = Patient Health Questionnaire; OHA = oral hypoglycemic agent; A1C = glycated hemoglobin.

**Table 4:** Logistic regression analyses of factors associated with depressive symptoms (*n* = 1529).

Variables	Univariate Analysis		Multivariable Analysis	
	OR (95% CI)	*p*	OR (95% CI)	*p*
**Sociodemographic variables**				
Sex				
Male	1		1	
Female	2.44 (1.56–3.84)	<0.001	1.22 (0.70–2.12)	0.490
Educational level (years)				
>12 (College and beyond)	1		1	
10–12 (High school)	2.30 (0.99–5.36)	0.053	1.45 (0.58–3.64)	0.424
7–9 (Middle school)	2.61 (1.10–6.23)	0.030	1.20 (0.47–3.05)	0.706
≤6 (Elementary school)	4.03 (1.94–8.39)	<0.001	1.29 (0.56–2.95)	0.551
Living				
With spouse	1		1	
Without spouse	2.90 (1.91–4.40)	<0.001	2.05 (1.30–3.24)	0.002
Employment				
Employed	1		1	
Unemployed	3.16 (1.94–5.14)	<0.001	2.05 (1.11–3.77)	0.021
Household income				
High	1		1	
Medium–high	1.621 (0.74–3.54)	0.224	1.67 (0.72–3.84)	0.230
Low–medium	2.69 (1.22–5.92)	0.014	1.87 (0.77–4.54)	0.169
Low	4.17 (1.96–8.86)	<0.001	2.95 (1.30–6.69)	0.010
**Health behavioral variables**				
Monthly drinking				
Yes	1		1	
No	1.88 (1.23–2.87)	0.004	1.30 (0.81–2.10)	0.280
Subjective health condition				
Good	1		1	
Fair	5.18 (1.15–23.34)	0.032	5.21 (1.05–25.90)	0.044
Poor	36.91 (8.68–156.99)	<0.001	19.07 (4.11–88.39)	<0.001
Perceived stress				
Low	1		1	
High	7.31 (4.67–11.46)	<0.001	6.98 (4.27–11.40)	<0.001
BMI (kg/m^2^)				
Normal (18.5 to 22.9)	1		1	
Underweight (<18.5)	0.46 (0.08–2.83)	0.403	0.11 (0.01–0.90)	0.059
Overweight (23.0 to 24.9)	0.71 (0.40–1.27)	0.253	0.23 (0.39–1.80)	0.236
Obesity (≥25.0)	0.92 (0.57–1.48)	0.731	0.84 (0.08–0.64)	0.282
**Diabetes-related variables**				
Diabetes duration (years)				
≤1	1		1	
2–5	2.11 (0.70–6.37)	0.183	2.64 (0.79–8.77)	0.120
6–10	2.31 (0.76–7.05)	0.140	2.36 (0.69–8.01)	0.169
11–20	2.15 (0.68–6.77)	0.192	2.42 (0.69–8.53)	0.169
>20	5.23 (1.65–16.57)	0.005	3.89 (1.09–13.95)	0.037
Management of diabetes				
Only OHA	1		1	
Only insulin	2.96 (0.86–10.20)	0.086	2.81 (0.68–11.62)	0.154
OHA and insulin	2.20 (1.07–4.54)	0.033	0.98 (0.35–2.74)	0.970
No medication	1.92 (0.21–17.28)	0.561	6.21 (0.71–54.31)	0.099
No treatment	1.39 (0.68–2.86)	0.365	1.21 (0.59–2.51)	0.604
**Comorbid diseases**				
Hypertension				
No	1		1	
Yes	1.67 (1.07–2.60)	0.025	1.30 (0.76–2.23)	0.330
Stroke				
No	1		1	
Yes	3.30 (1.80–6.06)	<0.001	1.94 (1.03–3.67)	0.041
Coronary heart disease				
No	1		1	
Yes	2.38 (1.40–4.06)	0.001	1.76 (0.96–3.23)	0.138

Note: CI = confidence interval; OR = odds ratio; *n* = sample size; BMI = body mass index; OHA = oral hypoglycemic agent.

**Table 5:** Multivariable logistic regression of factors associated with depressive symptoms by sex.

Variables	Female (*n* = 780)		Male (*n* = 749)	
	OR (95% CI)	*p*	OR (95% CI)	*p*
**Sociodemographic variables**				
Living				
With spouse	1		1	
Without spouse	1.74 (1.03–2.92)	0.038	3.22 (1.23–8.44)	0.018
Employment				
Employed	1		1	
Unemployed	1.83 (0.83–4.04)	0.136	3.19 (1.31–7.75)	0.011
Household income				
High	1			
Medium–high	2.45 (0.66–9.08)	0.180		
Low–medium	2.86 (0.77–10.57)	0.115		
Low	4.33 (1.22–15.37)	0.024		
**Health behavioral variables**				
Monthly drinking				
Yes	1			
No	1.84 (0.90–3.77)	0.093		
Subjective health condition				
Good	1		1	
Fair	3.51 (0.40–30.48)	0.254	7.52 (0.87–64.73)	0.066
Poor	16.76 (2.04–137.32)	0.009	25.75 (3.05–217.49)	0.005
Perceived stress				
Low	1		1	
High	4.69 (2.48–8.89)	<0.001	11.09 (4.95–24.88)	<0.001
BMI (kg/m^2^)				
Normal (18.5 to 22.9)			1	
Underweight (<18.5)			0.07 (0.01–0.69)	0.023
Overweight (23.0 to 24.9)			0.26 (0.09–0.71)	0.009
Obesity (≥25.0)			0.77 (0.36–1.64)	0.496
**Comorbid diseases**				
Hypertension				
No			1	
Yes			8.28 (2.72–25.21)	<0.001
Stroke				
No	1			
Yes	2.25 (1.03–4.91)	0.042		
Coronary heart disease				
No	1			
Yes	2.47 (1.14–5.38)	0.023		

Note: CI = confidence interval; OR = odds ratio; *n* = sample size; BMI = body mass index.

## Data Availability

Data are publicly available at https://knhanes.kdca.go.kr/knhanes/sub03/sub03_02_05.do (accessed on 13 August 2021).
